# Water photolysis effect on the long-term stability of a fiber optic hydrogen sensor with Pt/WO_3_

**DOI:** 10.1038/srep39160

**Published:** 2016-12-14

**Authors:** Xuexiang Zhong, Minghong Yang, Chujia Huang, Gaopeng Wang, Jixiang Dai, Wei Bai

**Affiliations:** 1 National Engineering Laboratory for Fiber Optic Sensing Technologies, Wuhan University of Technology, Wuhan, 430070, China; 2 Key Laboratory of Fiber Optic Sensing Technology and Information Processing, Ministry of Education, China

## Abstract

One of the technological challenges for hydrogen sensors is long-term stability and reliability. In this article, the UV-light irradiation was introduced into the hydrogen sensing process based on water photolysis effect of Pt/WO_3_. Ascribing to that, fiber optic hydrogen sensor with Pt/WO_3_ nanosheets as the sensing element was demonstrated with significantly improved performance of stability. Under UV irradiation, the hydrogen sensor exhibits higher sensitivity and resolution together with a smaller error range than that without UV irradiation. The enhanced performance could be attributed to the effective decomposition of water produced in the hydrogen sensing process due to the water photolysis effect of Pt/WO_3_. The influence of the water on stability was evaluated using experimental results, and the UV irradiation to remove water was analysed by theoretical and FT-IR spectra. This work provides new strategy of UV-light irradiation to promote the long-term stability of hydrogen sensor using Pt/WO_3_ as the sensing element.

With the increasing depletion of coal, oil and other fossil fuels, the development and utilization of new energy has become more important. As is well known, hydrogen is a clean and renewable energy that can be obtained from a wide variety of sources. Due to its status as the smallest molecule, hydrogen can easy leak in the processes of production, transportation, storage and utilization, and due to its flammability, it may explode strongly when its concentration is in a certain value (4–75%). For this reason, it is extremely important to explore the hydrogen sensors with reliable performance. Compared with conventional hydrogen sensors based on electrical characteristics, the fiber optic hydrogen sensor features excellent characteristics, including intrinsic safety, anti-electromagnetic interference and miniature design. Presently, there are many types of fiber optic hydrogen sensors, such as the interferometric sensor^1^,^2^, micro-mirror sensor^3^,^4^,^5^, evanescent sensor^6^,^7^,^8^,^9^,^10^ and fiber Bragg grating (FBG) sensor^11^,^12^,^13^,^14^,^15^,^16^. The FBG hydrogen sensor is especially suitable for distributed measurement and temperature compensation due to its wavelength multiplexing capability. Since the FBG hydrogen sensor is based on wavelength modulation, it can eliminate the influence of light power fluctuations and radically improve the anti-interference ability and stability (stable sensitivity during service period) of the sensor.

Tungsten trioxide (WO_3_) has drawn great research interest due to its special characteristics, including its capabilities of photocatalysis, and electrocatalysis, gasochromic and field-emission properties^17^,^18^,^19^,^20^. WO_3_ is an excellent metal oxide material for hydrogen gas sensing because of its chemical stability and gasochromic properties^20^,^21^. However, pure WO_3_ suffers from low sensitivity and a lack of selectivity. Doping with a noble metal, such as Au, Pt or Pd, is an effective means to enhance the detection of specific gases, as these metal catalysts increase the rate of interaction differently for different gases^4^,^5^,^10^,^12^,^22^. Among these metal catalysts, Pt is the most effective catalyst for sensing H_2_ and effectively increases the response and selectivity towards H_2_. Due to its fast response and high sensitivity, hydrogen sensors based on WO_3_ doped with Pt (Pt/WO_3_) are of intense interests and have been investigated extensively^12^,^15^,^16^,^21^,^23^. However, like most hydrogen sensors, degradation is still a severe problem for its application. Therefore, factors affecting the stability of the hydrogen sensor have also been widely investigated, such as water content^24^,^25^,^26^, catalyst poisoning^23^, and phase transition^27^. Among these factors, water content is regarded as an important factor in the long-term stability since water may cover the active Pt surface, resulting in catalyst poisoning and poor stability of the sensor^25^,^28^. Water can also decrease the sensitivity of the FBG sensors which is based on temperature sensing, because water can absorb the heat that is generated by the exothermic reaction between Pt/WO_3_ and H_2_^16^.

To improve the long-term stability and sensitivity of the fiber optic FBG hydrogen sensors, in this article, an UV-light irradiation strategy was introduced into the hydrogen sensing process based on the water photolysis effect of Pt/WO_3_^29^,^30^,^31^,^32^. To the best of our knowledge, while hydrogen sensors based on WO_3_ gasochromism have been developed and fabricated^12^,^15^,^16^,^21^,^23^, there have rarely been theoretical or experimental investigations of hydrogen sensors based on the both gasochromic and water photolysis properties of Pt/WO_3_. Through continual and systematic experimental tests over 3 months, the hydrogen sensor under UV irradiation exhibits significantly improved long-term stability, and the removal of water under UV irradiation was investigated and demonstrated through FT-IR measurement. The hydrogen sensing principle based on the gasochromism and water photolysis of Pt/WO_3_ is discussed.

## Results and Discussion

**Figure 1:**
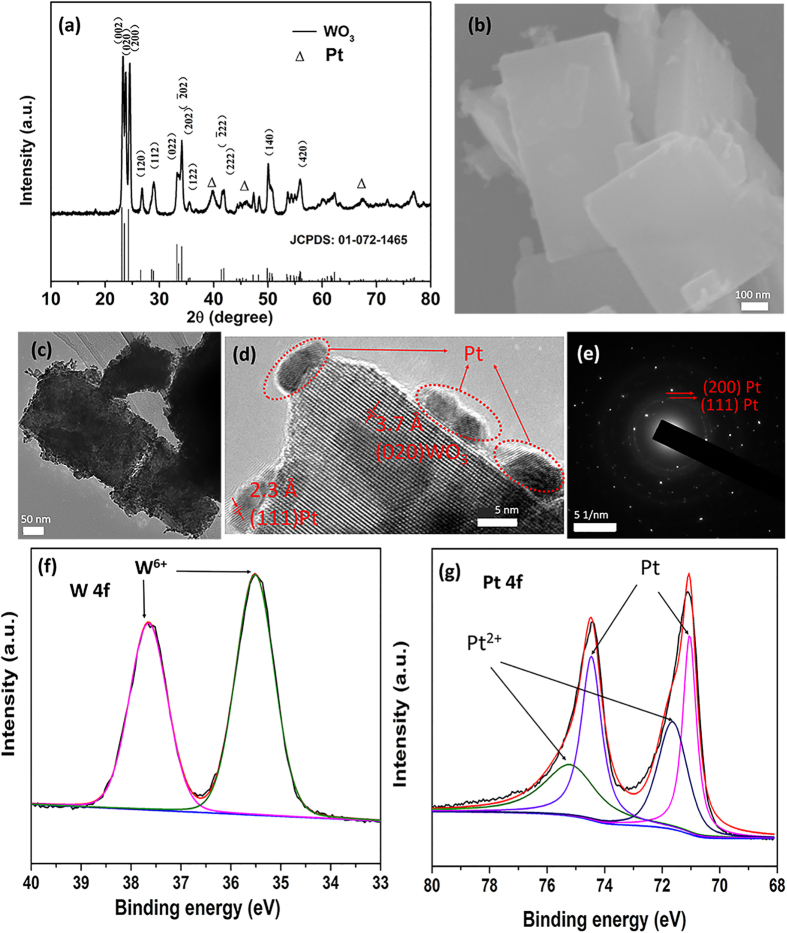
(**a**) XRD pattern; (**b**) FE-SEM image; (**c**) TEM image; (**d**) HRTEM image; (**e**) SADE pattern; (**f**,**g**) XPS spectra of Pt/WO_3_.

Pt/WO_3_ nanosheets were synthesized by a hydrothermal method followed by annealing. Figure 1(a) shows the XRD pattern of the Pt/WO_3_ nanosheets. The diffraction peaks agree with the monoclinic phase of WO_3_ with lattice parameters of a = 7.3 Å, b = 7.53 Å and c = 7.68 Å (JCPDS No. 01-072-1465). In addition, there are three diffraction peaks at 2θ = 39.82°, 46.31° and 67.50°, corresponding to the (111), (200) and (220) planes of the cubic phase of Pt (JCPDS No. 00-004-0802), respectively. The morphologies of Pt/WO_3_ are shown in Fig. 1(b), which exhibits that the WO_3_ was mainly composed of uniform nanosheets of approximately 430 nm × 780 nm, and the thickness is in the range of 50–90 nm. The morphology of the WO_3_ (Fig. S2), shows no significant change after Pt covering.

To obtain further insights into the morphology and structure of the Pt/WO_3_ nanosheets, TEM and HRTEM analysis were performed and the results are shown in Figs 1(c,d) and S2. It is clearly observed that the Pt particles effectively covered on the surface of WO_3_, and the particles size of the Pt is approximately 5–10 nm, which results in the weak peaks of Pt shown in the XRD pattern. The HRTEM image of the Pt/WO_3_ nanosheets shows the typical WO_3_ and Pt lattice fringes. The contact between the WO_3_ and Pt called ohmic contact, will facilitate electron transfer from the Pt to the WO_3_ during the photoexcitation, resulting in enhanced charge separation and photocatalytic efficiency. The periodic fringe spacings of ~3.7 and ~2.3 Å agree well with the interplanar spacing of the (020) plane of monoclinic WO_3_ and the (111) plane of cubic Pt, respectively. The selected-area electron diffraction (SAED) pattern (Fig. 1(e)) reveals the crystalline nature of the Pt/WO_3_ nanosheets, as also indicated by their XRD result. To obtain the surface chemical composition and state of the WO_3_ nanosheets, we performed high-resolution XPS analysis. The XPS spectra of Pt/WO_3_ are presented in Fig. 1(f) and (g). In the W4f XPS spectrum, the only pair of peaks located at 35.5 and 37.7 eV corresponds to the W4f_7/2_ and W4f_5/2_ of W^6+^. In the Pt4f levels of the samples, the strong doublet decomposition peaks at 71.1 and 74.5 eV could be assigned to metallic Pt, while the weak doublet decomposition peaks at 71.7 and 75.3 eV were corresponded to Pt(II) oxide, such as PtO^22^,^33^. The peak area of metallic Pt was much larger than that of the Pt(II) oxide, which indicated that most of the Pt was in the metallic state^34^.

**Figure 2:**
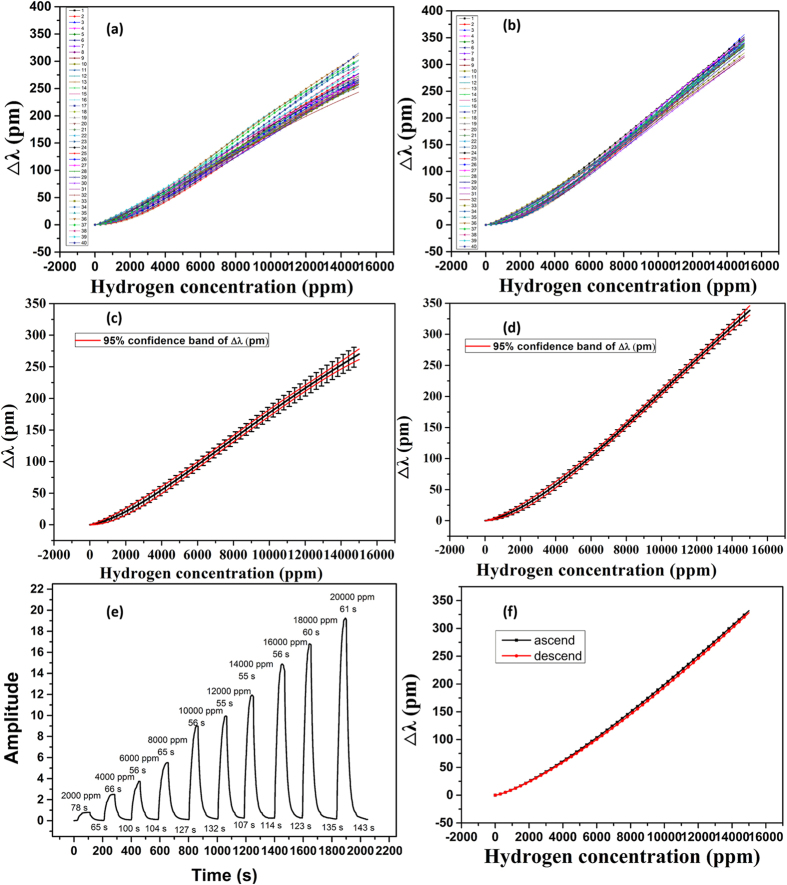
Sensitivity curve for different H_2_ concentrations (**a**) without UV irradiation; and (**b**) with UV irradiation; (**c**) error analysis of (**a**); (**d**) error analysis of (**b**); (**e**) time response of sensor in (**b**); (**f**) wavelength shifts of (**b**) under different H_2_ concentration in both ascending and descending conditions.

The sensing probe was placed in a sealed chamber for the hydrogen sensing performance calibration. Hydrogen gas at various concentrations was injected into the sealed chamber, and the shifted FBG wavelengths were recorded. The experimental data were fitted by Equation (7) and the linearity was greater than 0.99 (Fig. S3). Subsequently, the cycling stability of the coatings under two different hydrogen sensing process (with and without UV irradiation) was compared. At room temperature, hydrogen concentration was controlled from low to high by a flowmeter and the characteristics of the sensor probes with and without UV irradiation were tested once every two days. To verify the water effect on the sensing stability, experiments were conducted for 40 cycles. The experimental result of the stability test is shown in Fig. 2. 40 cycles were plotted to determine the stability. From Fig. 2(a) and (b), it can be found that in the case of UV irradiation, the cycle stability is better than that without UV irradiation. The error bars and 95% confidence band in Fig. 2(c) and (d) represent the range that were obtained for each sensor after forty cycles. As shown in Fig. 2(c) and (d), it can be concluded that the FBG characteristic wavelength shift with (without) UV irradiation reaches 380 pm (270 pm) at hydrogen concentration of 15000 ppm, which means the average H_2_ sensitivity is 0.023 pm/ppm (0.018 pm/ppm). Given the resolution of the fiber optic spectrometer is 1 pm, the equivalent H_2_ concentration resolution of the sensor is calculated to be 43.5 ppm (56 ppm). And the greatest discrepancy of the sensor in the relative wavelength shift is ±9.5 pm (±16 pm), which corresponds to ±410.9 ppm (±888.9 ppm) of hydrogen concentration. The minimum error of sensor in the relative wavelength shift is ±0.025 pm (±0.04 pm) which corresponds to ±1.1 ppm (±2.2 ppm) of hydrogen concentration.

From the comparison, it can be concluded that the sensitivity, resolution and stability of the sensing probe with UV irradiation are higher than that those without UV irradiation. These performance properties were attributed to the water decomposition on the surface of the Pt/WO_3_ by UV irradiation. Figure 2(e) displays the response time of the Pt/WO_3_ with UV irradiation under different H_2_ concentrations. It can be deduced that the response time is approximately 60s and the recovery time is less than 150s. The sensor thus presents a good response at room temperature.

The wavelength shifts of the proposed sensor with UV irradiation under different H_2_ levels in both the ascending and descending condition are shown in Fig. 2(f). It is clear that the two curves are well consistent with each other at various H_2_ concentrations. However, a small of discrepancy can be seen, and this discrepancy increases with the H_2_ concentration. The greatest discrepancy was found to be ±5 pm, which is reasonable compared with the greatest discrepancy in Fig. 2(d). It should be noted that the time interval between the 35th and 36th hydrogen detection is over one month, which indicates that the sensor shows excellent reversibility and stability.

**Figure 3:**
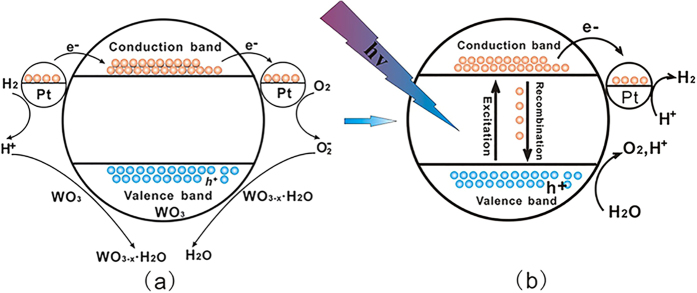
Schematic illustration of Pt/WO_3_ (**a**) react with H_2_ and O_2_; (**b**) UV water photolysis.

To explain the water generation process and understand the water decomposition under UV irradiation during the sensing process, a mechanism based on gasochromic and photocatalytic process is proposed and shown in Fig. 3. In a system containing WO_3_ and Pt, since the work function of Pt (5.1 eV) is smaller than that of WO_3_ (5.7 eV)^35^, a deflexion of the energy band of WO_3_ occurs due to the effect of the ohmic contact (Fig. S4). Consequently, Pt exhibits an excess positive charge, while WO_3_ accumulates excess e^−^. When H_2_ is injected into the chamber, Pt dissociates the adsorbed H_2_ molecules into ions and traps the produced electrons (e^−^) as an electron pool due to the deflexed energy band. Meanwhile, the WO_3_ is transformed to unstable compounds WO_3−x_ · xH_2_O under the environment of H^+^.

Subsequently, the color of the WO_3_ changes to blue. This is the coloration process of WO_3_ and the reaction can be expressed as the following equation:


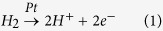



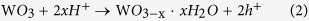


Multi e^−^ trapped by the Pt are then used for the further reduction of oxygen molecules. This leads to the increased generation of O_2_^−^ and thereby unstable compounds of WO_3−x_ · xH_2_O under the environment of h^+^ that can be oxidized into WO_3_. This is the bleaching process of WO_3_ and the reaction can be expressed as the following equation:


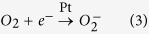



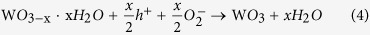


When UV light is irradiated (hv) onto the Pt/WO_3_ system, electrons (e^−^) are generated in the conduction band (CB) and holes (h^+^) are generated in the valence band (VB). These photo-induced holes likely remained in the VB of WO_3_, whereas electrons are trapped in the CB of Pt, resulting in an efficient space separation of photo-induced charge carriers. The holes stored in the VB of the WO_3_ are used for splitting H_2_O molecule in the air near the surface of the WO_3_ into H^+^ and O_2_, and the produced H^+^ is then transferred to the surface of the Pt and reduced to H_2_ by absorbing e^−^. The major steps in this mechanism under UV light irradiation are summarized in the following reactions^29^,^36^:


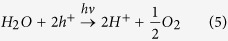



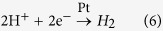


**Figure 4:**
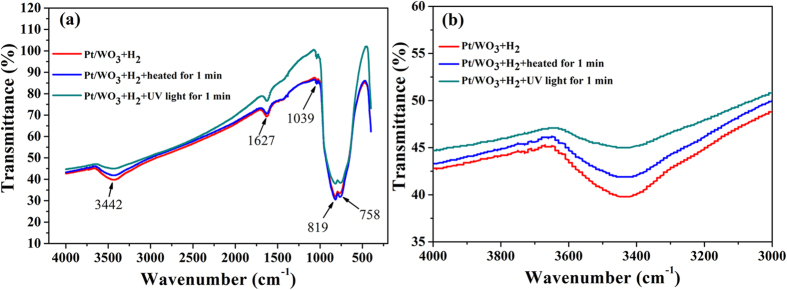
(**a**) FT-IR spectra of Pt/WO_3_; (**b**) Enlargement of peak at 3442 cm^−1^.

To verify the water photolysis effect of the UV irradiation on the decomposition of water, the sample reacted with H_2_ was characterized by FT-IR after irradiated by UV and heated at 120 °C for 1 min respectively. The FT-IR spectra are shown in Fig. 4. The intense broad band observed at 3442 cm^−1^ and 1627 cm^−1^ are due to the O-H stretching vibrations and bending vibrations of H_2_O, respectively^36^. The peak at 1039 cm^−1^ corresponds to the W-OH bending vibration mode^37^, while the absorption peaks at 819 cm^−1^,and 758 cm^−1^ are assigned to the O-W-O bonds of WO_3_^36^. It can be observed that the peak intensity of the 3442 cm^−1^ significantly reduced under UV irradiation for 1 min, while the peak intensity is only weakened slightly after heating at 120 °C for 1 min, indicating that the strategy of UV irradiation has distinct superiority for the remove of the water. FT-IR results of the samples irradiated with UV light for different time were obtained (Fig. S5). There is a distinct decreasing trend of the peak intensity at 3442 cm^−1^ as the time increase, indicating the gradual removal of water.

Based on the results and analysis above, the UV-light irradiation is an effective method to eliminate the water produced during the hydrogen sensing process. Ascribing to that, the stability and sensitivity of the hydrogen sensor under UV irradiation are greatly improved.

## Conclusions

In summary, Pt/WO_3_ nanosheets have been successfully fabricated and deposited on an FBG for hydrogen detection. Different from previous reports, an UV-light irradiation strategy was introduced into the hydrogen sensing process to eliminate the produced water based on the water photolysis effect of Pt/WO_3_. Ascribing to that, the hydrogen sensor under UV irradiation exhibits significantly improved long-term stability through experimental tests over 3 months. The sensor also shows higher sensitivity (0.023 pm/ppm) and resolution (43.5 ppm) with UV irradiation than that without UV irradiation. Through the FT-IR spectra, the UV irradiation on the decomposition of the water produced in the hydrogen sensing process is confirmed. This study provides a new strategy of UV-light irradiation to promote the long-term stability of a hydrogen sensor with Pt/WO_3_ as the sensing element.

## Methods

### Synthesis

WO_3_ · H_2_O nanosheets were synthesized by a hydrothermal method as reported in a previous work^38^. In a typical process, 0.66 g of sodium tungstate (Na_2_WO_4_ · 2H_2_O) and 0.4 g of citric acid were mixed and dissolved into 40 mL of distilled water under 10 min of magnetic stirring to form a transparent solution. Then the aqueous solution was slowly acidified to a pH range of 1–1.2 with 10 mL of 3 mol/L HCl. Subsequently, a yellow precipitate was obtained, and 0.8 g oxalic acid was added into solution. After 20 min of stirring, the solution was transferred into a 50-mL Teflon-lined stainless steel autoclave, that was sealed and heated at 160 °C for 12 h, and then cooled to room temperature naturally. The obtained precipitates were separated by centrifugation, washed several times with distilled water and absolute ethanol and then dried in air at 60 °C for 4 h. After that, 1 mmol of the as-synthesized WO_3_ · H_2_O and 0.2 mmol acetyl acetone platinum (Pt(acac)_2_) were mixed. The mixture was grinded sufficiently and annealed in air at 315 °C at a heating rate of 10 °C/min for 2 h to obtain Pt/WO_3_ nanosheets.

### Characterization

The phase of the Pt/WO_3_ was investigated by X-ray diffraction (XRD) using a Bruker D8 Advance X-ray diffractometer with Cu Kα X-ray source. The morphology was characterized by using a Zeiss Ultra Plus field emission scanning electron microscope (FE-SEM). Transmission electron microscopic (TEM), high resolution transmission electron microscopic (HRTEM) images and selected area electron diffraction (SAED) pattern were collected using a JEOL JEM-2100F STEM/EDS microscope. FT-IR spectra were obtained with Thermo Scientific Nicolet 6700. The oxidation state and relative chemical composition of Pt/WO_3_ were evaluated by X-ray photoelectron spectroscopy (XPS) in a Kratos AXIS-ULTRA DLD-600 W.

### Fabrication of sensor probe

The sensor device was fabricated by uniformly coating the as-synthesized Pt/WO_3_ powder mixed with an appropriate amount of de-ionized water on the FBG fiber, and the FBG fiber was fixed on a glass substrate with a groove so that the Pt/WO_3_ coating could be stably immobilized on the section of the FBG grating.

### Hydrogen sensing characterization

**Figure 5:**
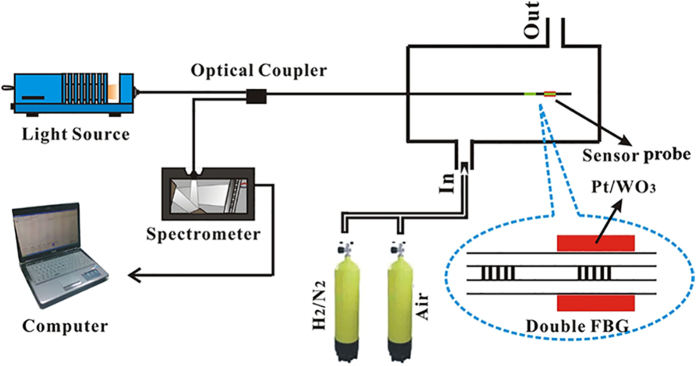
Configuration of FBG hydrogen sensor.

Figure 5 shows a schematic of the configuration of the FBG hydrogen sensing characterization system. The system consists of a tunable light source, a 3-dB optical fiber coupler, a sensing probe and a customized optical spectrum analyzer with a resolution of 1 pm. The sensing probe consists of two FBGs, one employed for temperature compensation, and other FBG coated with Pt/WO_3_ detect hydrogen as the sensing probe. The hydrogen sensing performance test was conducted at room temperature with nitrogen as the carrier gas (with an ambient 50% RH). Mixture of nitrogen and hydrogen with variable H_2_ concentrations were provided by a Beijing Sevenstar Electronics CS200 flowmeter with a resolution of 1 ppm. The hydrogen sensing process was divided into two stages: (i) the sensor probe was exposed to a mixture of nitrogen and hydrogen with a variable H_2_ concentrations and the spectrum of the tested FBG with Pt/WO_3_ was measured and (ii) after that, the sample was exposed to air along with (or without) ultraviolet light (λ = 365 nm) irradiation for 1 min at 8 W/cm^2^ using a EXFO LX300 smart LED point light curing equipment to decompose the water that was generated in stage (i). The relationship between the center wavelength of the FBG and the temperature change has been reported in our previous work^15^, and the FBG theory based on the Pt/WO_3_ coatings can be described as:


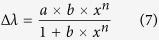


where “a” is related to the hydrogen media sensing material and “b” and “n” are constants of reactions. A detail derivation of Equation (7) was presented in the Supplementary Information.

## Additional Information

**How to cite this article:** Zhong, X. *et al*. Water photolysis effect on the long-term stability of a fiber optic hydrogen sensor with Pt/WO_3_. *Sci. Rep.* **6**, 39160; doi: 10.1038/srep39160 (2016).

**Publisher’s note:** Springer Nature remains neutral with regard to jurisdictional claims in published maps and institutional affiliations.

## Supplementary Material

Supplementary Information
