# Supplementary material for: Water photolysis effect on the long-term stability of a fiber optic hydrogen sensor with Pt/WO3
Source: Sci Rep. 2016 Dec 14;6:39160. doi: 10.1038/srep39160 (PMC5155222; doi:10.1038/srep39160)
Supplement: Supplementary Information [file srep39160-s1.docx]

**Water photolysis effect on the long-term stability of a fiber optic hydrogen sensor with Pt/WO_3_**

**Xuexiang Zhong^1^, Minghong Yang*^1,2^, Chujia Huang*^1^, Gaopeng Wang^1^, Jixiang Dai^1^, Wei Bai^1^**

^1^ National Engineering Laboratory for Fiber Optic Sensing Technologies, Wuhan University of Technology, Wuhan, China, 430070

^2^ Key Laboratory of Fiber Optic Sensing Technology and Information Processing, Ministry of Education, China

[**minghong.yang@whut.edu.cn**](mailto:minghong.yang@whut.edu.cn)**；**[**huangchuj@whut.edu.cn**](mailto:huangchuj@whut.edu.cn)

**
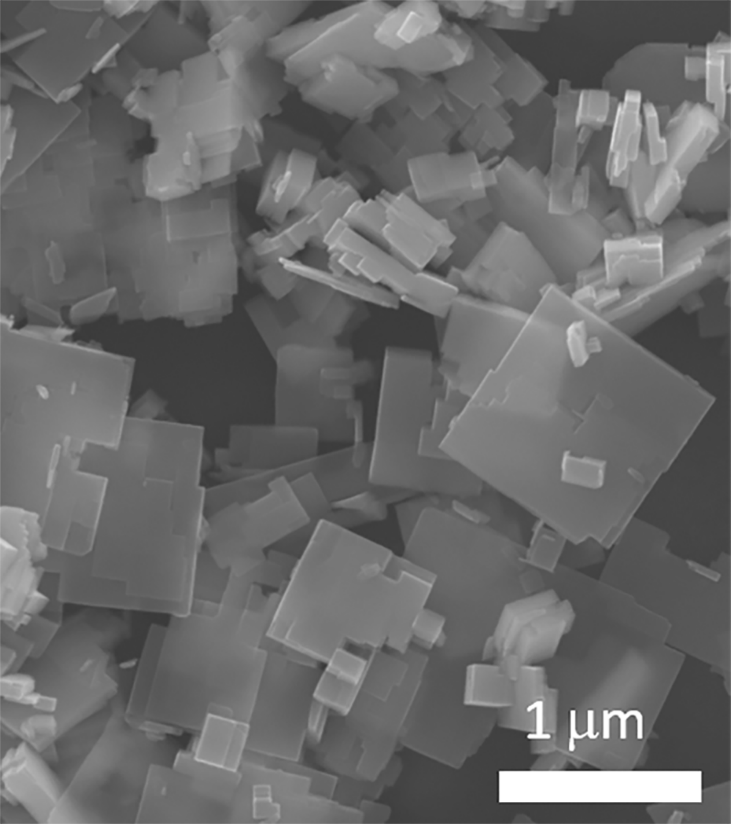
**

Figure S1 SEM image of WO_3_.


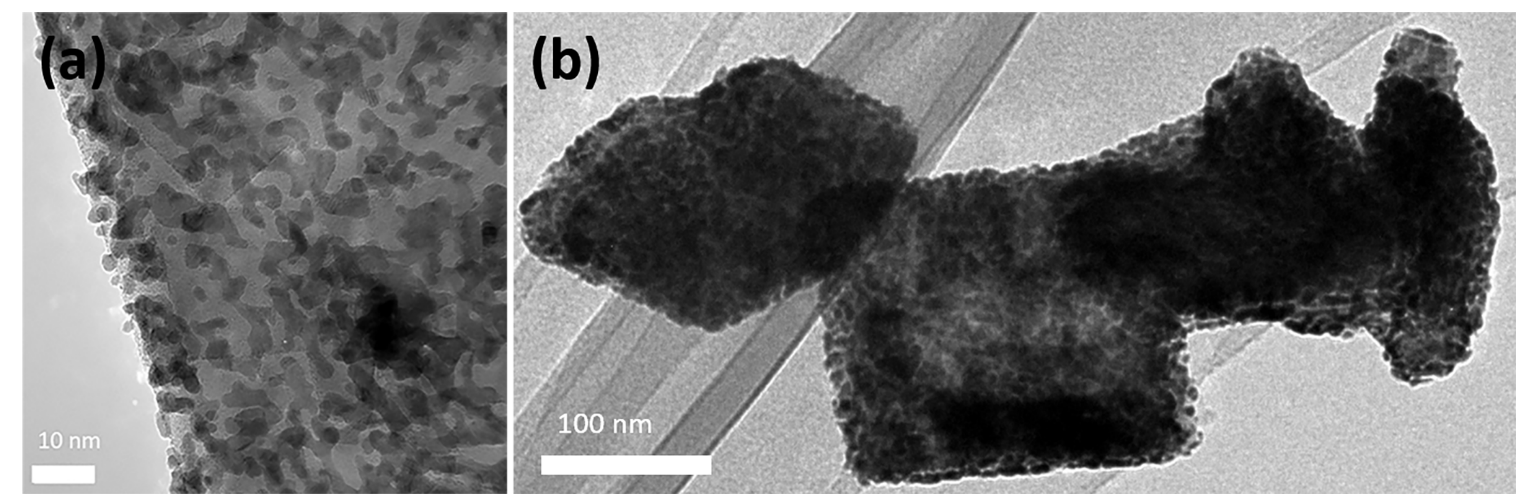


Figure S2 TEM image of Pt/WO_3_.


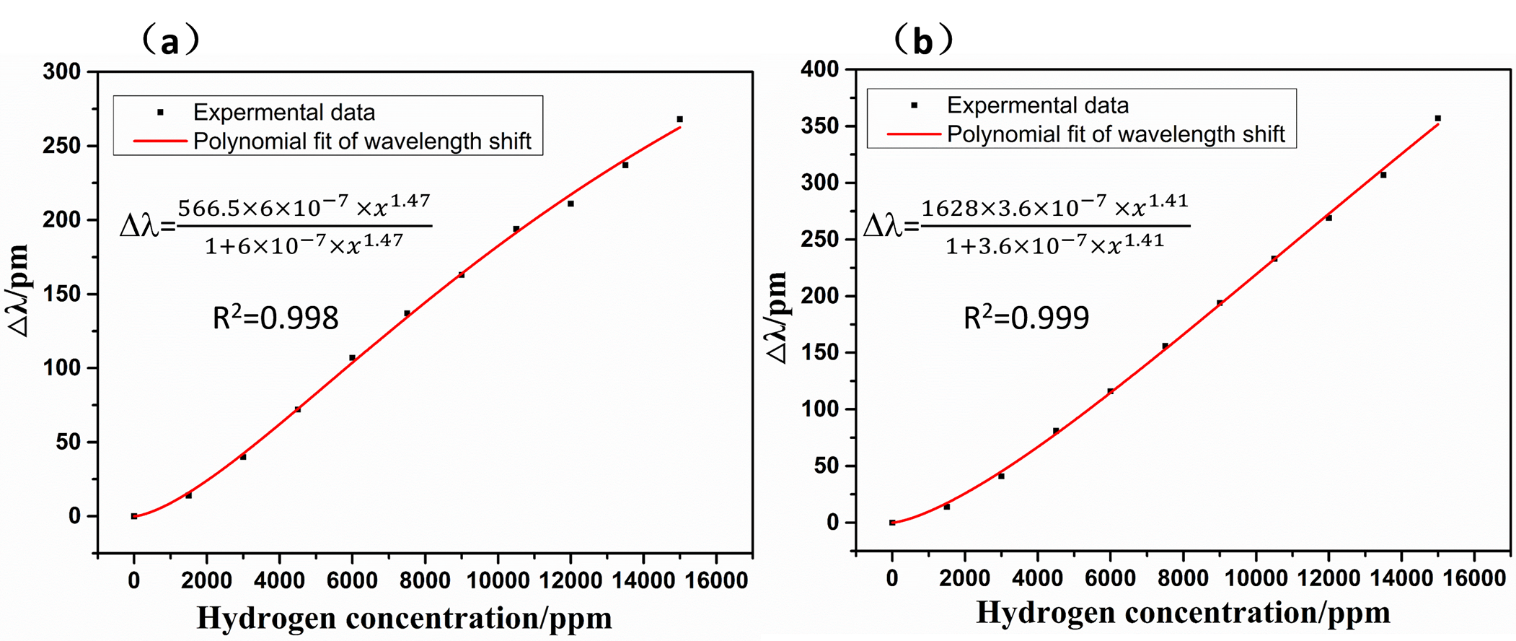


Figure S3 The shift of wavelength to different hydrogen concentrations (a) without UV irradiation; (b) with UV irradiation.


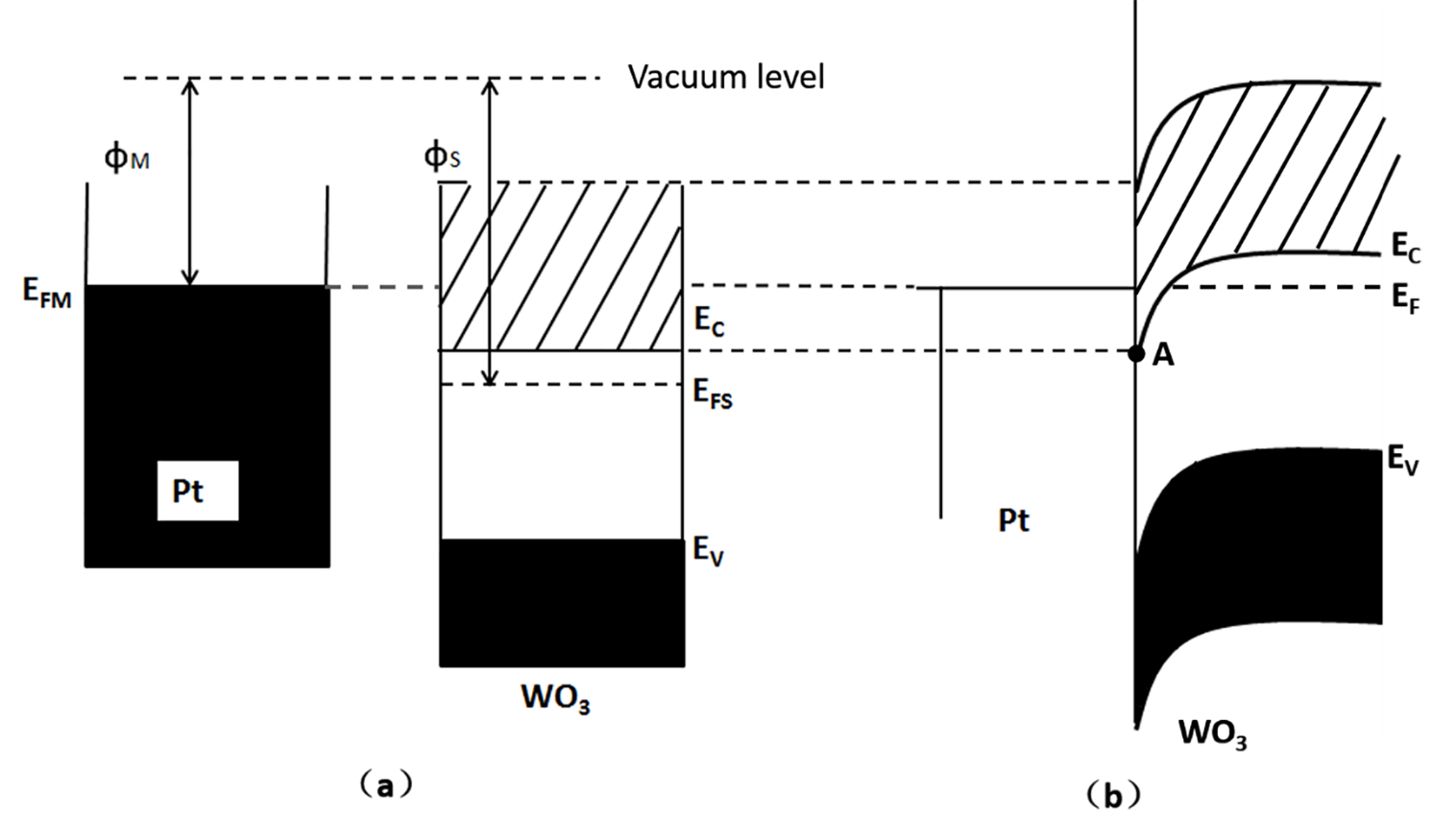


Figure S4 The ohmic contact of Pt/WO_3_ (a) before contact; (b) after contact.


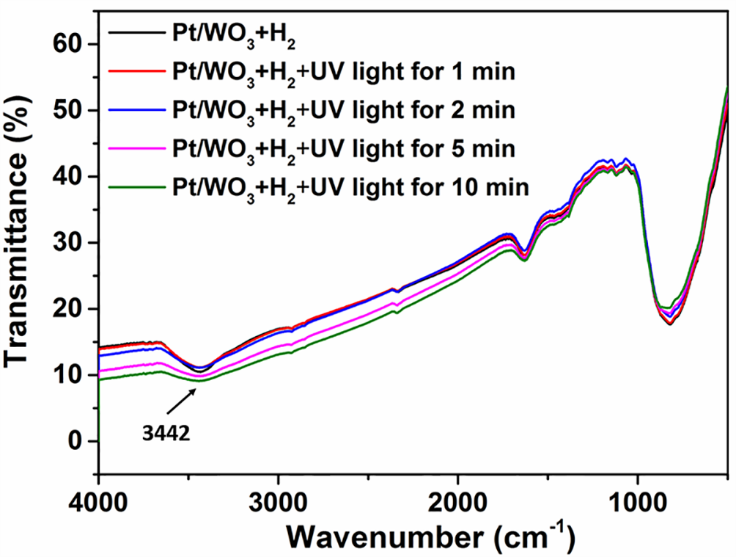


Figure S5 FT-IR spectra of Pt/WO_3_ irradiated with UV light for different times.

Detailed derivation of Equation (7)$\Delta\lambda\text{=}\frac{a\times b\times x^{n}}{1+b\times x^{n}}$:

Not considering the interference of the external environment on the strain, the effect of the temperature on the center wavelength of the FBG is

$\frac{\Delta\lambda}{\lambda}=\left[\alpha+\xi+(1-P_{e})(\alpha_{s}-\alpha)\right]\Delta T$ (1)

$\alpha$——thermal expansion coefficient of optical fiber

$\xi$——thermo-optical coefficient of optical fiber

$P_{e}$——effective elastic-optic constant of optical fiber

$\alpha_{s}$——thermal expansion coefficient of substrate

where$P_{e}=\frac{n_{eff}^{2}}{2}\left[P_{12}-\upsilon(P_{11}+P_{12})\right]$

$n_{eff}$——effective refractive index

$P_{11}$，$P_{12}$——elastic-optic constants of optical fiber

$\upsilon$——Poisson's ratio

For a reactive substance on the surface of a cylinder, the heat changes from the hydrogen absorption are:

$2\pi rlQ_{ts}-2\pi rl\omega(T-T_{0})=c\pi r^{2}l\rho\frac{dT}{dt}$ (2)

$Q_{ts}$——formation heat of reaction per unit of time

$\omega$——heat transfer efficiency, which is a constant for a fixed structure

$T_{0}$——ambient temperature

$c$,$r$,$l$,$\rho$——specific heat, radius, length and density of the cylinder, respectively.

With the boundary initial conditions of t=0，T=T_0_, it can be obtained that

$T=T_{0}+\frac{Q_{ts}}{\omega}\left[1-\exp(\frac{-2\omega t}{cr\rho})\right]$ (3)

When the time tends to infinity,

$T_{\max}=T_{0}+\frac{Q_{ts}}{\omega}$ (4)

$\Delta T=\frac{Q_{ts}}{\omega}$ (5)

$Q_{ts}$ is connected to the generation rate of water

$Q_{ts}=f_{1}\frac{dH_{2}O}{dt}$ (6)

For the system of Pt/WO_3_, the gasochromic process can be described as follows:

$$H_{2}\to^{Pt} 2H^{+}+2e^{-}$$

$$\text{W}O_{3}+2xH^{+}\to\text{W}O_{3-\text{x}}\bullet xH_{2}O+2h^{+}$$

$$O_{2}+e^{-}\to^{\text{Pt}} O_{2}^{-}$$

$$\text{W}O_{3-\text{x}}\bullet\text{x}H_{2}O+\frac{x}{2}h^{+}+\frac{x}{2}O_{2}^{-}\to\text{W}O_{3}+xH_{2}O$$

Simplified below with the reaction rate equation of each step：

（fast）$r_{1}=k_{1}\Gamma_{H_{2}}=k_{-1}\Gamma_{H}^{2}$
（slow）$r_{2}=k_{2}\Gamma_{O}\Gamma_{H}$
（fast）$r_{3}=k_{3}\Gamma_{OH}\Gamma_{H}$

$\Gamma_{O}$，$\Gamma_{H}$，$\Gamma_{OH}$，$\Gamma_{H_{2}}$——reaction concentration of each substance.

From chemical kinetics, it is known that the total rate of this sequential reaction is equal tot hat of the slowest step, so the total rate of the reaction is

$r=\frac{dH_{2}O}{dt}\approx r_{2}=k_{2}\Gamma_{O}\Gamma_{H}=k_{2}\Gamma_{O}\left(\frac{k_{1}}{k_{-1}}\Gamma_{H_{2}}\right)^{\frac{1}{2}}$ (7)

With the Arrhenius equation, the relationship between the reaction rate and the ambient temperature can be quantitatively described：

$$k=Ae^{-E_{a}/RT}$$

A——apparent frequency factor

E_a_——activation energy

R——ideal gas constant

When the reaction reaches equilibrium，$k_{1}\approx k_{-1}$，and then Equation (7) can be simplified to

$r\approx A\Gamma_{O}\left(\Gamma_{H_{2}}\right)^{\frac{1}{2}}e^{-E_{a}/RT}$ (8)

Supposing the saturated adsorption amount is$\Gamma_{m,H_{2}}^{a}$, and the equilibrium adsorption amount is$\Gamma_{H_{2}}^{a}$, and with the general formula of isotherm adsorption：

$\Gamma_{H_{2}}^{a}=\frac{\Gamma_{m,H_{2}}^{a}v_{1}C_{H_{2}}\left(\frac{1}{n}+v_{2}C_{H_{2}}^{n-1}\right)}{1+v_{1}C_{H_{2}}\left(1+v_{2}C_{H_{2}}^{n-1}\right)}$ (9)

$v_{1}$——first-stage adsorption coefficient

$v_{2}$——second-stage adsorption coefficient

$n$——adsorption number

Due to the strong adsorption ability of precious metals, such as Pt, to H, it is assumed that the adsorption type is multimolecular adsorption, and the adsorption of level one is far inferior to that of level two, i.e.,$n>1$，$v_{1}C_{H_{2}}<<1$，and hence Equation (9) can be simplified to

$\Gamma_{H_{2}}^{a}=\frac{\Gamma_{m,H_{2}}^{a}vC_{H_{2}}^{n}}{1+vC_{H_{2}}^{n}}$ (10)

where$v=v_{1}v_{2}$

When the reaction reaches equilibrium, then by combining with Equations (8) and (10), it is obtained that

$r=\frac{f_{2}BC_{H_{2}}^{\frac{n}{2}}}{1+BC_{H_{2}}^{\frac{n}{2}}}\cdot e^{-E_{a}/RT}$ (11)

where$f_{2}=A\Gamma_{O}\left(\Gamma_{m,H_{2}}^{a}\right)^{\frac{1}{2}}$ and $B=v^{\frac{1}{2}}$

Taking full account of Equations (1), (5), (6) and (11), it is obtained that

$\Delta\lambda=\frac{fBC_{H_{2}}^{\frac{n}{2}}}{1+BC_{H_{2}}^{\frac{n}{2}}}\cdot e^{-E_{a}/RT}$ (12)

where$f=\frac{\left[\alpha+\xi+(1-P_{e})(\alpha_{s}-\alpha)\right]\lambda f_{1}f_{2}}{\omega}$

Equation (12) is the formula for the hydrogen adsorption reaction of a sensing probe with a certain amount of Pt/WO_3_, in which the influence of the coating material was not considered. If the factors of the sensing material are considered, a material influence factor δ is added to Equation (12), and then the complete sensing formula is obtained:

$\Delta\lambda=\delta\cdot\frac{fBC_{H_{2}}^{\frac{n}{2}}}{1+BC_{H_{2}}^{\frac{n}{2}}}\cdot e^{-E_{a}/RT}$ (13)

Considering that the performance was tested at room temperature, T is a constant. In this condition, Equation (13) can be expressed as

$\Delta\lambda\text{=}\frac{a\times b\times x^{n}}{1+b\times x^{n}}$ (14)

where “a” is related to the hydrogen media sensing material and “b” and “n” are constants of reaction.
